# The Development and Optimization of Hot-Melt Extruded Amorphous Solid Dispersions Containing Rivaroxaban in Combination with Polymers

**DOI:** 10.3390/pharmaceutics13030344

**Published:** 2021-03-06

**Authors:** Jong-Hwa Lee, Hyeong Sik Jeong, Jong-Woo Jeong, Tae-Sung Koo, Do-Kyun Kim, Young Ho Cho, Gye Won Lee

**Affiliations:** 1 Bioanalysis and Pharmacokinetic Research Group, Korea Institute of Toxicology, Daejeon 35365, Korea; jhl@kitox.re.kr (J.-H.L.); egaria1105@naver.com (J.-W.J.); 2 Department of Pharmaceutics & Biotechnology, Konyang University, Daejeon 35365, Korea; goodmanu@naver.com; 3 Graduate School of New Drug Discovery and Development, Chungnam National University, Daejeon 35365, Korea; kootae@cnu.ac.kr; 4 Korea Zoonosis Research Institute, Jeonbuk Natinal University, Iksan 54531, Korea; dkkim714@gmail.com

**Keywords:** rivaroxaban (RXB), amorphous solid dispersion (ASD), hot-melt extruding technique, quality by design (QbD), bioavailability

## Abstract

Rivaroxaban (RXB), a novel oral anticoagulant that directly inhibits factor Xa, is a poorly soluble drug belonging to Biopharmaceutics Classification System (BCS) class II. In this study, a hot-melt extruded amorphous solid dispersion (HME-ASD) containing RXB is prepared by changing the drug:polymer ratio (Polyvinylpyrrolidione-vinyl acetate 64, 1:1–1:4) and barrel temperature (200–240 °C), fixed at 20% of Cremophor^®^ RH 40 and 15 rpm of the screw speed, using the hot-melt extruding technique. This study evaluates the solubility, dissolution behavior, and bioavailability for application to oral drug delivery and optimizes the formulation of rivaroxaban amorphous solid dispersion (RXB-ASD). Based on a central composite design, optimized RXB-ASD (PVP VA 64 ratio 1:4.1, barrel temperature 216.1 °C, Cremophor^®^ RH 40 20%, screw speed 15 rpm) showed satisfactory results for dependent variables. An in vitro drug dissolution study exhibited relatively high dissolution in four media and achieved around an 80% cumulative drug release in 120 min. Optimized RXB-ASD was stable under the accelerated condition for three months without a change in crystallinity and the dissolution rate. A pharmacokinetic study of RXB-ASD in rats showed that the absorption was markedly increased in terms of rate and amount, i.e., the systemic exposure values, compared to raw RXB powder. These results showed the application of quality by design (QbD) in the formulation development of hot-melt extruded RXB-ASD, which can be used as an oral drug delivery system by increasing the dissolution rate and bioavailability.

## 1. Introduction

Rivaroxaban (RXB), an anticoagulant agent and the active ingredient in Xarelto^®^ tablets, is used to treat deep vein thrombosis (DVT) and pulmonary embolism (PE). RXB does not require a cofactor for activity since RXB inhibits free factor Xa (FXa) and prothrombinase activity [1] compared to anticoagulant drugs such as vitamin K and warfarin that are not widely used in the clinic due to their toxic side effects. However, RXB is classified as a Biopharmaceutics Classification System Class II (BCS Class II) drug and it has low solubility (20 μg/mL) in aqueous solutions. There have been several attempts in the preparation of polymeric amorphous solid dispersions (ASDs) containing RXB [2,3]. To improve the solubility, various techniques such as liposomes, nanosuspensions, solid dispersions, and cyclodextrin inclusions were tested [4,5,6,7]. Solvent evaporation, freeze-drying, supercritical fluid method, spray drying, and thermal melting are applied in the manufacture of solid dispersion systems [8,9,10,11,12,13,14,15]. Hot-melt extrusion (HME), used for ASDs of poorly water-soluble active pharmaceutical ingredients (APIs), is a complex commercialized technique. In comparison to traditional methods of preparation of ASDs, HME is the most promising solvent-free, continuous, industry feasible, and scalable process for preparation of ASDs [16]. It is possible to improve the stability by preventing hydrolysis and oxidation and reducing residence times at high temperatures and screw rotation. During the preparation of such ASDs, the API is usually mixed/extruded with a molten of thermos-softened polymer.

Recently, many studies have been conducted on methods of preparing physically stable ASDs using polymer mixtures and the QbD approach, which allows for enhancing pharmaceutical development through design efforts from product development conceptualization to its commercialization [17,18,19,20,21,22]. The design of experiments (DoEs) for the drug development and product process is classified into screening design, factorial design, and response surface methodology (RSM). RSM is used to estimate possible effects, quadratic effects, the shape of the response surface, and interactions, and is positively applied to pharmaceutical development because there can be a variation of only one parameter at a time, keeping other parameters constant, although two or more variables can be studied simultaneously [23,24]. In addition, RSM has the advantage in terms of reduced process variability, higher percentage yields, lower treatment time, and cost-effectiveness. It estimates the relative significance of different variables.

However, a systemic attempt using the QbD to improve the solubility of RXB has not been reported in HME-based ASDs, although there are several approaches of preparation of polymeric ASDs containing RXB [2,3]. Therefore, HME-based ASD on RXB using optimal polymer combination was developed in terms of improvement of the dissolution behavior and oral bioavailability, based on the experimental design method of RSM. 

## 2. Materials and Methods

### 2.1. Materials

RXB (purity > 98.5%; Hanseo Chemical, Pyeongtaek, Korea) and Xarelto^®^ tablets (20 mg, Bayer, Luverkusen, Germany) were purchased. 

Polyvinylpyrrolidone-vinyl acetate 64 (PVP VA 64, Kollidone^®^ VA 64), Soluplus^®^ (Polyvinyl caprolactam-polyvinyl acetate-polyethylene glycol graft co-polymer), Cremophor^®^ RH 40 (PEG-40 hydrogenated castor oil), polyvinylpyrrolidone K 90 (PVP 90), Solutol^®^ HS 15 (Polyoxyl 15-hydroxystearate), and Kolliphor^®^ 188 (Poloxamer 188) were obtained from BASF (Ludwigshafen, Germany). Polyvinylalcohol (PVA, MW 89,000–98,000) was purchased from Sigma (St. Louis, MO, USA), and polyethylene glycol (PEG) was purchased from Daejung (Seoul, Korea). Gelucire^®^ 44/14 (Lauroyl polyoxy-32-glycerides) and Gelucire^®^ 50/13 (Stearoyl macrogol-32 glycrides) were obtained from Gattefosse (Saint-Priest, France). Carboxymethyl cellulose (CMC) and hydroxypropyl cellulose (HPC) were purchased from Samchun (Pyeongtaek, Korea). Acetonitrile and methanol for HPLC were purchased from J.T. Baker (Seoul, Korea). All the other chemicals and reagents used were of analytical grade.

### 2.2. Before the Study 

#### 2.2.1. Selection of a Carrier

To study critical material attributes (CMA), first of all, a 1% aqueous solution containing various polymers and surfactants was prepared, and we added various amounts of RXB to determine how much of it dissolved in the solution. The mixtures were stirred continuously for 72 h at 25 ± 0.5 °C and centrifuged at 15,000 rpm for 5 min; then, the supernatants were filtered through membrane filters (0.45 μm, Whatman, PA, USA). The filtrates were diluted in solution (chloroform: methanol = 1:7 (*v*/*v*, %), and the concentration of RXB was quantified. Next, by changing the concentration of various polymers and surfactants in aqueous solution from 0.5% to 10% (*w*/*w*, i.e., 0.5%, 1%, 2%, 5%, and 10%), the maximum concentration of RXB that could be dissolved was determined.

The concentration of RXB was analyzed by an HPLC (Shimadzu, Japan) system; the system featured an LC10-AD isocratic pump, a SPD-10A VP variable spectrophotometric detector, and a Shimpak GIS ODS column (5 μm pore diameter; 4.6 × 150 mm, Shimadzu, Japan). An acetonitrile/water mixture (55/45, *v*/*v*, %) served as the mobile phase; the flow rate was 1.2 mL/min, and the detection wavelength was 250 nm. The RXB calibration curve was linear (r = 0.9999) over the concentration range of 0.625–200 µg/mL.

#### 2.2.2. HME Process Condition

The solubility and dissolution of RXB-ASDs were selected as critical process parameters (CPP) in HME technology. RXB-ASDs were prepared by alternating the barrel temperatures between 140 °C, 180 °C, 200 °C, 220 °C, and 240 °C and the screw speeds between 10, 15, and 20 rpm for a Haake Mini CTW hot-melt extruder (Thermo Scientific Inc., Waltham, MA, USA) fixed at a weight polymer ratio of 1:4 (RXB: Soluplus^®^). The effect of screw speed on solubility was observed by adjusting the screw speed to 10, 15, or 20 rpm with the temperature fixed at 200 °C.

**Figure 1:**
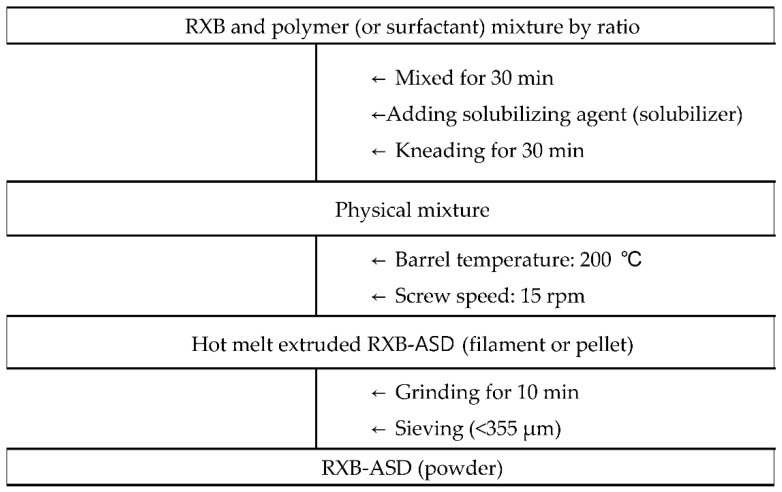
Preparation process of hot-melt extruded RXB-ASD.

Next, after fixing the temperature and screw speed, the effects on the solubility and dissolution at 6 h in SGF (pH 6.8) of RXB-ASDs, prepared as shown in Figure 1 with various polymer compositions (Table 1), were evaluated.

#### 2.2.3. Preformulation Study of RXB-ASDs Using a Full Factorial Design (FFD)

Based on the results of failure mode effect analysis (FMEA), with a preformulation experimental design with a hot-melt extruder at a 15 rpm screw speed, the effect of independent variables (PVP VA 64 ratio (X1, 1:1, 1:2, 1:4), total weight of Cremophor^®^ RH 40 (X2, 0, 10, 20 *w*/*w*%) and barrel temperature (X3, 180, 200, 220 °C) on dependent variables (content (Y1) and the dissolution rate at 6 h in SIF (Y2)) was monitored (Table 2).

### 2.3. Optimization by Central Composite Design (CCD)

Based on preformulation results, the RXB-ASD experimental design and data analysis were conducted using the central composite design module in Minitab version 18 software (Minitab Inc., State College, PA, USA). The RSM central composite design method was used to optimize critical factors at 20% of Cremophor^®^ RH 40 and 15 rpm screw speed. The effect of the independent variables such as the polymer ratio (X1, 1:2–1:4) and barrel temperature (X2, 200–220 °C) on the dependent variables such as the content (Y1) and dissolution rate at 2 h (Y2) and 6 h (Y3) in SGF (pH 1.2) and the dissolution rate at 2 h (Y4) and 6 h (Y5) in SIF (pH 6.8) was evaluated.

The linear equation of the model is as follows:

Y = A0 + A1·X1 + A2·X2 + A3·X1·X1 + A4·X2·X2 + A5·X1·X2,

where Y is the response of the dependent variables associated with each factor-level combination; A0 is the intercept; A1, A2, A3, A4, and A5 are the regression coefficients; and X1 and X2 are the independent variables.

The data were fitted to a second-order polynomial equation, and regression coefficients were obtained. Analysis of variance (ANOVA) was conducted to evaluate the significance and adequacy of the developed regression model. The adequacy of the response surface models was clarified by the determination coefficient (*R*^2^) and the lack of fit.

One-way ANOVA and multiple regression analysis were performed to test the significance of the model and factor coefficients. The polynomial, plots, and two-contour plots also revealed the interactions between each independent variable.

After generating the polynomial equations relating to the dependent and independent variables, optimization of the dependent variables (Y1, Y2, Y3, Y4, and Y5) was performed using a desirability function to obtain the levels of X1 and X2 that maximized the dependent variable.

### 2.4. Physicochemical Evaluation of RXB-ASD

The calorimetric responses of samples were recorded on a differential scanning calorimetry (DSC) system (N-650, Scinco, Korea) equipped with a refrigerated cooling system, calibrated for temperature and heat flow using a high-purity indium standard. The sample cell was purged with dry nitrogen at a flow rate of 40 mL/min. The 3–5-mg samples were laid on crimped aluminum pans and measured at a heating rate of 20 °C/min up to 250 °C.

Powder X-ray diffraction (PXRD patterns of different samples were recorded at room temperature using a Smartlab X-ray diffractometer (Rigaku Mechatronics Co. Ltd., Tokyo, Japan) equipped with a 2θ compensating slit, using Cu Kα radiation (1.5406 Å) at 45 kV and 200 mA passing through a nickel filter with an IS slit (0.5°), a soller slit (5°), and receiving slit (20 mm). Samples were mounted on a zero-background sample holder and subjected to a continuous scan over a Bragg angle 2 θ range of 2–35° at a step size of 0.01° and scan rate of 5°/min. Obtained diffractograms were analyzed with the PDXL processing program.

To monitor the intermolecular interaction between drug and polymers, the crystalline RXB and ASD were monitored with FT-IR by a conventional KBr pellet method using an FTIR-4100 spectrophotometer (JASCO, Tokyo, Japan).

The surface morphology of powder samples was monitored under a scanning electron microscope (SEM) (S-3400, Hitachi, Ltd., Tokyo, Japan) with an excitation voltage of 25 kV. The powder samples were mounted onto a steel stage and sputter-coated with gold using ion sputtering (E-1010, Hitachi, Ltd.) prior to analysis.

### 2.5. Drug Content, In Vitro Dissolution Test, and Food Effect

For quantification of the drug content, RXB-ASDs containing 2 mg of RXB were dissolved in 2 mL of dimethyl sulfoxide. After stirring for 3 h, the solution was filtered with a membrane and diluted 100-fold (0.1 mL of filtrate in a total volume of 10 mL) in the solution (chloroform: methanol = 12.5:87.5, *v*/*v*%). The concentration of RXB in the diluted solution was quantified using HPLC.

Dissolution was tested using a dissolution tester (DRS-14, Labbindia, India) with 900 mL distilled water (DW), SGF (pH 1.2), acetate buffer (pH 4.0), and SIF (pH 6.8), via agitation with a paddle at 75 rpm and 37 ± 0.5 °C.

The optimized RXB-ASD sample, placed within a hard gelatin capsule (size number 2) containing equivalent amounts of RXB (20 mg) in a sinker, was placed in a dissolution medium, and 5-mL aliquot samples were withdrawn at certain time intervals (0.10, 30, 60, 120, 240, and 360 min) and filtered using a membrane filter (0.45 μm, Whatman, PA, USA). The filtered samples were diluted with a chloroform and methanol mixture (1:7 *v*/*v*%), and the concentration of the drug was determined by HPLC. The release rates were compared with those of a conventional tablet (Xalretol^®^, 20 mg as RXB), with RXB powder as a negative control.

To investigate the effect of food on optimized RXB-ASD, the dissolution rate was monitored for fasted-state simulated gastric fluid (FaSSGF), fed-state simulated gastric fluid (FeSSGF), fasted-state simulated intestinal fluid (FaSSIF), and fed-state simulated intestinal fluid (FeSSIF).

The similarity of release profiles between the test preparation and reference preparation in different dissolution media was evaluated as the similarity factor, *f*_2_. The relevant equation is as follows [24,25]:

$$f_{2}=50\times \log\left\{{\left[1+\frac{1}{n}\sum_{i=1}^{n}{\left|R_{j}-T_{j}\right|}^{2}\right]}^{-0.5}\times 100\right\}$$

where *n* is the number of sampling time points, and *R* and *T* represent the cumulative dissolution of the drug at the specified time point in the respective reference formulation and the test formulation. The value of *f*_2_ ranged from 0 to 100; when the value exceeded 50, the drug release profiles between the reference formulation and test formulation were considered to be similar.

### 2.6. Stability

To check the stability, hard gelatin capsules (size number 2) filled with RXB-ASD were submitted to accelerated degenerative conditions (40 °C/75% RH). The appearance, drug content, and in vitro dissolution rates were evaluated for three months.

### 2.7. Application to Pharmacokinetic Study

RXB and RXB-ASD were administered orally at dose of 10 mg/kg to elucidate the absorption in male Sprague–Dawley rats aged seven weeks and weighing 199–225 g (Orient Bio, Seongnam, Korea). In addition, to monitor the bioavailability, RXB-ASD was administered intravenously at dose of 2 mg/kg. For all experiments, the animals were kept in plastic cages with free access to a standard rat diet (PMI Nutrition International, Richmond, IN, USA) and water at a temperature of 20–26 °C, with a 12 h light–dark cycle and a relative humidity of 40–60% under the guidance of the Institutional Animal Care and Use Committee of Chungnam National University (202003A-CNU-055, 23 June and 8 August 2020, Daejeon, Korea).

Prior to dosing, animals were fasted for 14 h and provided with free access to water after a further 4 h. RXB was homogenized in normal saline and administered for oral administration at a volume of 5 mL/kg and solubilized in mixture composed of 10% DMSO, 40% PEG 400, and 50% normal saline for intravenous injection at a volume of 2 mL/kg. Blood samples (300 μL) were obtained from the jugular vein at 0.0167, 0.33, 1, 2, 4, 8, 12, and 24 h after oral dosing and at 0.083, 0.25, 0.5, 1, 2, 5, 8, and 24 h after intravenous dosing in four animals per group. The blood samples were immediately centrifuged at 17,600× g for 5 min, and the separated plasma samples were stored at −20 °C until analysis. 

With respect to RXB in rat plasma, the bioanalytical method for RXB in rat plasma was adjusted and optimized based on a previously established method [26]. LC used a 1200 series system from Agilent Technologies (Santa Clara, CA, USA) composed of a binary pump, degasser, autosampler, and column oven. A Zorbax phenyl column (50 × 2.1 mm, 5 µm particle size; Agilent, Santa Clara, CA, USA) was used with the mobile phase consisting of (A) 10 mM ammonium formate containing 0.1% formic acid of total volume in water (pH 4.5) and (B) methanol with gradient elution at a 0.3 mL/min flow rate. Samples (2 µL) were analyzed using the following isocratic mode for 3 min with a composition of 40% A and 60% B. The temperatures of the column oven and autosampler were maintained at 40 °C and 10 °C, respectively.

MS was performed on the API 4000 Qtrap LC-MS/MS system (AB Sciex, Framingham, MA, USA) operated in the negative ion mode. The ion source parameters were set as follows: curtain gas 20 psi, ion spray voltage 5500 V, ion source temperature 600 °C, nebulizing gas (GS1) 60 psi, drying gas (GS2) 50 psi. The MS parameters of declustering potential and collision energy for RXB were optimized at 86 V and 37 V, respectively, and those for the internal standard were optimized at 81 V and 25 V, respectively. The ion transitions in multiple-reaction monitoring (MRM) were monitored at *m*/*z* 436.2→145.0 for RXB and *m*/*z* 338.2→296.1 for linezolid, an internal standard. The data were acquired using Analyst (version 1.4.2) from AB Sciex.

The pharmacokinetic analysis was performed by a noncompartmental analysis using Phoenix WinNonlin^®^ 8.1 (Pharsight Corp., Cary, NC, USA). The peak plasma concentration (C_max_) and the time to reach the peak concentration (T_max_) were obtained directly from the profile of the time‒plasma concentration. The elimination rate constant (K_el_) was determined by linear regression in the terminal phase. The half-life (T_1/2_) in the terminal phase was calculated by dividing ln 2 by the K_el_. In addition, we determined the systemic clearance (CL), the volume of distribution (V_d_), and mean residence time (MRT). The area under the plasma concentration‒time curve from time zero to infinity (AUC_inf_) was calculated via the linear trapezoidal rule and the standard area extrapolation method [27].

## 3. Results and Discussion

### 3.1. Before the Study

#### 3.1.1. Selection of Carrier

CMA and CPP were selected by FMEA. The CMAs were the type and ratio of the polymer, plasticizer, and polymer combination, and the CPPs were the barrel temperature and screw speed.

The components used should solubilize the drug and ensure continuing solubility in the ultimate dispersion. The solubility of RXB in distilled water is 0.09 ± 0.00 μg/mL, which makes it a very poorly soluble drug. Among the polymers, Cremophor^®^ RH 40 showed high-solubilizing capacities for RXB and was 30.33 ± 5.24 μg/mL. However, most polymer solutions, except for HPC and HPMC, had values less than 10 μg/mL (Table 3).

**Figure 2:**
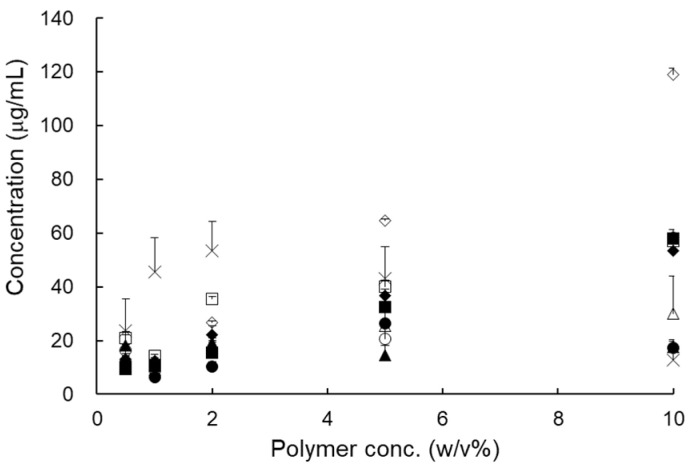
Solubility of RXB according to the aqueous polymer solution concentration (*n* = 3, means ± SD). (Key: ■: Gelucire^®^ 44/14, □: Gelucire^®^ 50/13, ◆: Solutol^®^ HS 15, ▲: PEG 4000, △: PEG 20,000, ○: PVP VA 64, ●: PVP K 90, ×: PVA, ◇: Soluplus^®^.

The solubility of RXB, according to the polymer concentration, was significantly increased in Soluplus^®^ and PVP VA 64 (Figure 2). However, the solubility increased by 2% and then decreased in Gelucire^®^ 50/14 and PVA. Finally, based on these results, a combination of PVP VA 64 and Cremophor^®^ RH 40 (or Soluplus^®^) was selected for the preparation of RXB-ASDs using HME technology.

#### 3.1.2. HME Process Conditions

In general, to prepare thermodynamically stable ASD using hot-melt extrusion, high temperatures above the melting point of API are avoided [28]. Therefore, the processing temperatures were selected based on T_g_ of the blend of the polymeric system (i.e., 180–240 °C) and a melting point of API (i.e., 228–234 °C) to obtain well-solidified extrudates.

When RXB-ASDs were prepared with various barrel temperatures and screw speeds, the solubility increased as the barrel temperature increased, but was not affected by the screw speed (data not shown).

When RXB-ASDs were prepared at 200 °C barrel temperature and 15 rpm screw speed, the content of RXB was approximately 90–100% except for the PVA formulation (F6 and F9). This is thought to be due to the relatively high viscosity of PVA.

The dissolution rate was higher in formulations with the polymer mixture than in the polymer alone. In the formulations of F9 and F10 with PVP VA 64 and Cremophor^®^ RH 40 (or PVA), the dissolution rates were 80.80 ± 1.16 and 83.29 ± 0.33%, respectively (Table 1). By comparison, the formulation of F4, which used the polymer alone, had a rate of 51.89% and the value increased by approximately 160%, which indicated that PVP VA 64 and Cremophor^®^ RH 40 (or PVA) can be recommended to increase the dissolution rate.

#### 3.1.3. Preformulation Study of RXB-ASDs by FFD

Considering the thermodynamic stability of API, the critical process factors were selected as follows: 200–240 °C barrel temperature, 1:1~1:4 PVP VA 64 ratio, and 0–20% Cremophor^®^ RH 40 based on the total weight.

After evaluating the contents and dissolution rate of the RXB-ASDs, prepared according to the FFD, multiple regression analysis was performed (data not shown). That is, the content was approximately 90–105% and was not significantly affected by the independent variables. However, the dissolution rate was significantly affected by the ratio of PVP VA 64 and the barrel temperature (*p* < 0.05) and increased sharply over 1:2 and 200 °C. In addition, the dissolution rate increased by more than 15% with Cremophor^®^ RH 40, but this result was not significant; it had a significant interaction with the PVA VA64 barrel temperature. Therefore, in the present study, to obtain solid and free-flow powder, the content of Cremophor^®^ RH 40 was set to 20% (*w*/*w*) of the total weight.

### 3.2. Optimization by CCD

**Figure 3:**
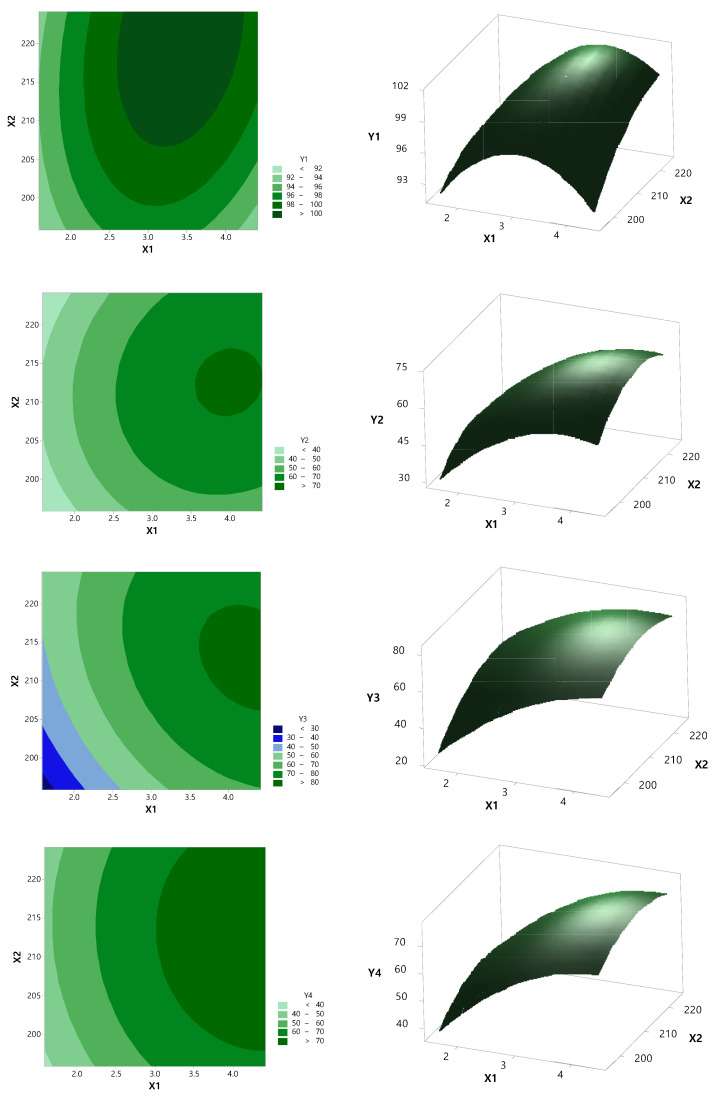

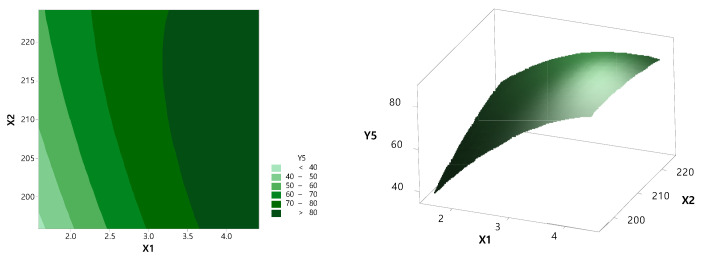
Contour and 3D response surface plot of dependent variables against independent variables: X1, PVP VA64 ratio; X2, barrel temperature (°C); Y1, content (%); Y2, dissolution rate (%) in SGF (2 h); Y3, dissolution rate (%) in SGF (6 h); Y4, dissolution rate (%) in SIF (2 h); Y5, dissolution rate (%) in SIF (6 h).

**Figure 4:**
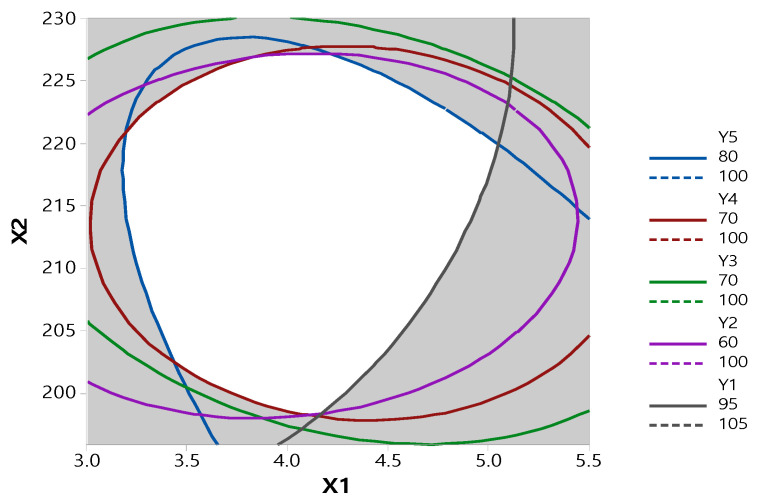
Nesting contour plot of dependent variables versus independent variables for all responses.

Based on the results of the preformulation study in Section 3.1.3, the independent variables were the ratio (X1) of PVA VA 64 and the barrel temperature (X2). The results of the dependent variable for the central synthesis design and the regression variance analysis for the quadratic term for the model fit (*p* < 0.05) and the multiple correlation coefficients are shown in Table 4 and Table 5. In addition, the response surface method and contour plot are shown in Figure 3 and Figure 4.

**Figure 5:**
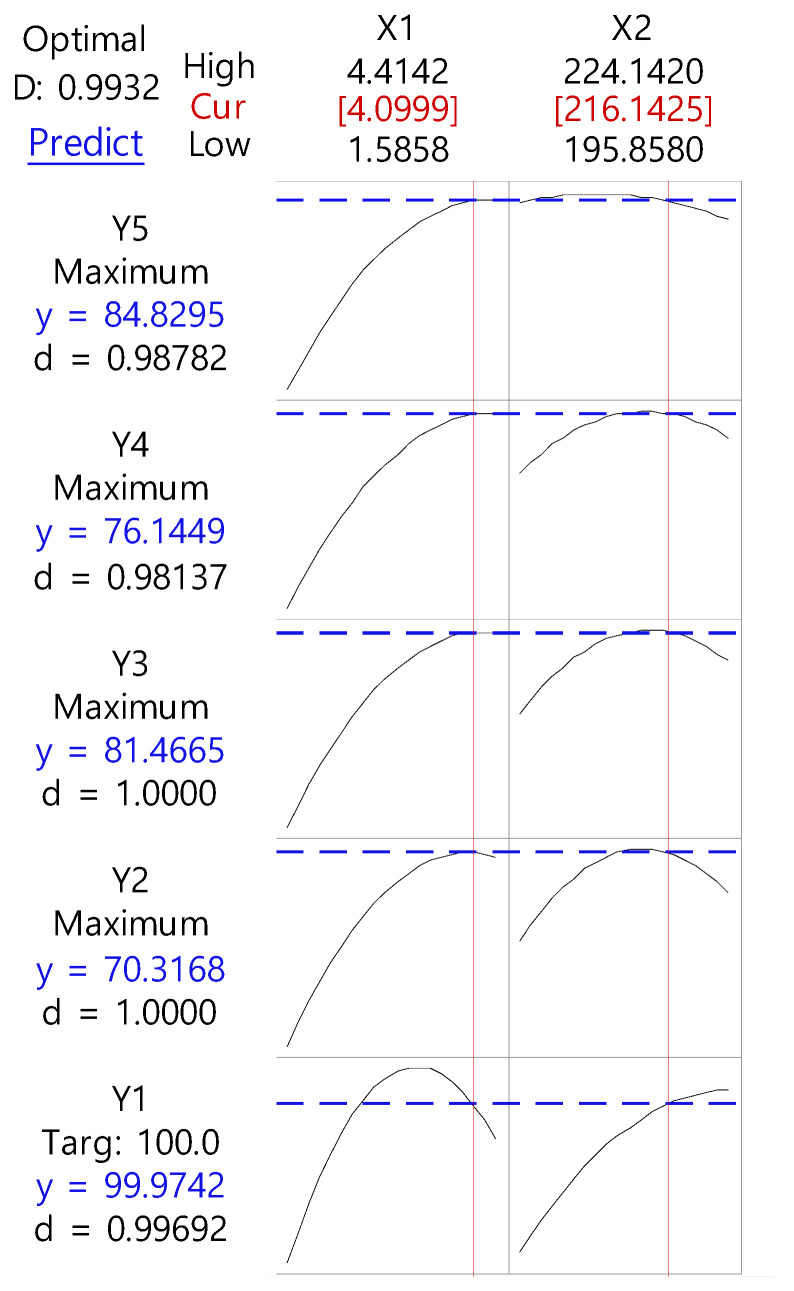
Response optimization profile of dependent variables for independent variables of hot-melt extruded RXB-ASD.

As the ratio of PVP VA 64 increased, the dissolution rate increased normally, then steeply to 1:3, and then became slower. This result is consistent with Pawar’s study [29]. The enhancement in the dissolution rate of RXB-ASD may be due to the amorphous state drug that offers a lower thermodynamic barrier to dissolution. The glassy state of RXB-ASD yielded the highest solubility and dissolution rate because the drug is molecularly dispersed in a polymer mixture system resulting in the formation of an amorphous system. Other different factors that might contribute to the increase in the dissolution rate of RXB are increased wettability and hydrophilicity, improved dispersibility, and reduced particle size of the drug. In addition, as the barrel temperature increased to 215 °C, the dissolution rate increased, but it decreased above 220 °C. The contour plot at a content of 95–105% and also 60–70% for 2 h and 70–80% for 6 h of dissolution is shown in Figure 5. The ratio of RXB to PVP VA 64 and the barrel temperature were set to 1:4.099 and 216.1425 °C, respectively, to show the optimum dissolution rate at SGF (pH 1.2) or SIF (pH 6.8), which indicated that the values had a high prediction ability (99.32%).

### 3.3. Physicochemical Evaluation of RXB-ASD

**Figure 6:**
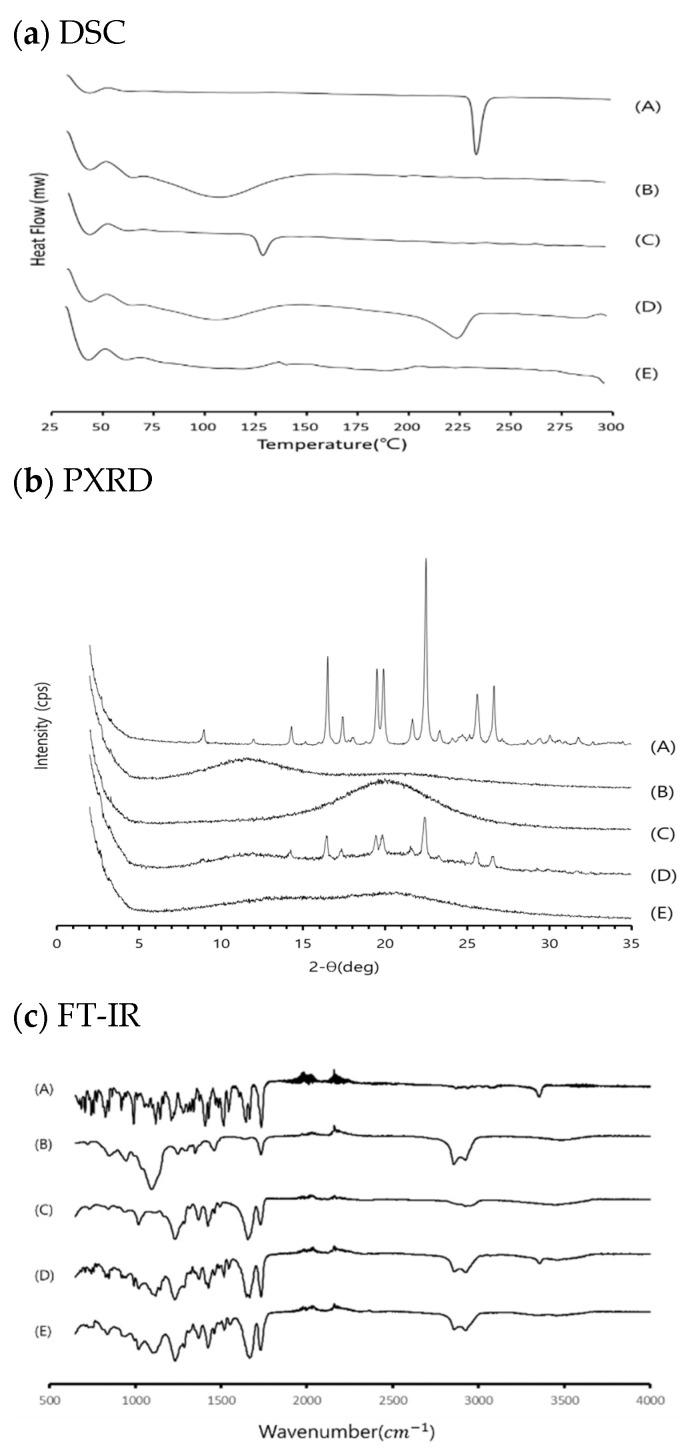
DSC (**a**), PXRD (**b**), and FT-IR (**c**) profiles of samples: (A) RXB, (B) PVP VA64, (C) Cremophor^®^ RH 40, (D) physical mixture, and (E) optimized RXB-ASD.

The DSC spectrum of RXB showed a sharp endothermic peak at 232.92 °C, which corresponds to its melting point. The distinct characteristic peak of RXB observed in the thermogram of the physical mixture signifies the existence of crystalline RXB. A shift (224.17 °C) in the characteristic endotherm of RXB was noticed along with a slightly reduced peak intensity which can be attributed to the partial interaction of RXB with PVP VA 64 (Figure 6a). However, the characteristic endotherm of RXB completely disappeared in optimized RXB-ASD, indicating an amorphous or molecular dispersion state. The formation of a disordered molecular structure or a reduction in the particle size of the drug can occur since the binding force between the drug molecules was weakened in the mixture of the drug and the polymer [30]. 

Analysis of powder diffraction patterns of RXB showed a distinct crystalline phase with characteristic sharp peaks, confirming the polymorphic form I of RXB. The diffractogram of physical mixtures showed distinct characteristics peaks for RXB with/without reduced peak intensity, suggesting the existence of crystalline RXB in physical mixtures (Figure 6b). All the polymers showed a small and broad endotherm of moisture in DSC and a halo pattern in PXRD. Optimized RXB-ASD was found to be stable and showed the absence of crystallinity as evidenced by DSC and PXRD. 

**Figure 7:**
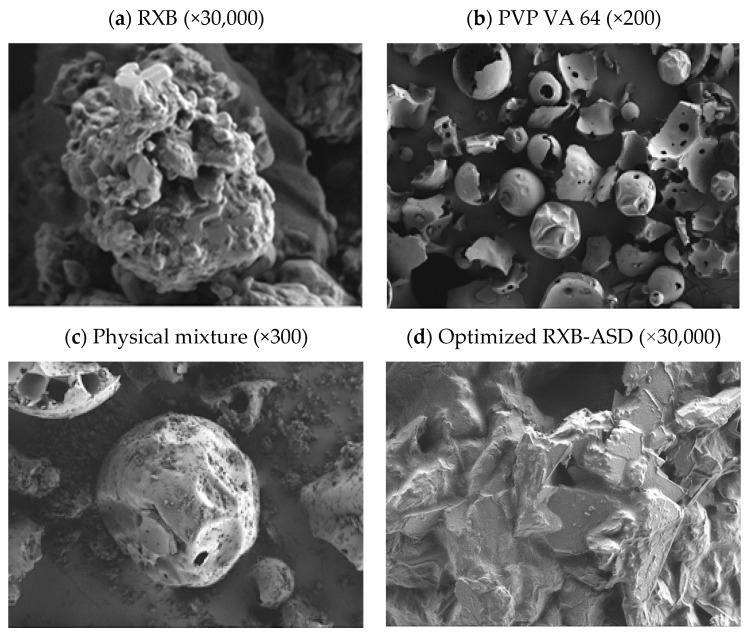
SEM images of samples: (**a**) RXB; (**b**) PVP VA64; (**c**) physical mixture; (**d**) optimized RXB-ASD.

The FT-IR spectra of samples (RXB, PVP VA 64, Cremophor^®^ RH 40, and RXB-ASD) at 500–4000 cm^−1^ wavenumber are stacked for visual analysis in Figure 7c. RXB was identified by characteristic peaks at 3353 cm^−1^ (N–H stretch), 1735 cm^−1^ (C=O stretch), and 1590 cm^−1^ (Ar‒Cl stretch). On the whole, the spectrum of the RXB-ASD showed no significant differences from excipients, which indicated that no new chemical bonds were created in RXB-ASD and proved there was good compatibility between the drug and excipients.

SEM analysis of ASD is widely used to probe the particle morphology after the formation of amorphous dispersions, conducted to acquire the photomicrographs for RXB, polymers, and optimized RXB-ASD. RXB consists of a mixture of large and small particles deposited with microparticles, which could be due to micronization or any other size reduction processes at the time of manufacturing (Figure 7). The PVP VA 64 has spherical particles with a smooth surface. RXB-ASD showed morphologies consisting of a collapsed and corrugated surface without crystallinity, indicating that the binding force between molecules was reduced and finally increased the solubility and dissolution rate.

### 3.4. In Vitro Dissolution Test and Food Effect

**Figure 8:**
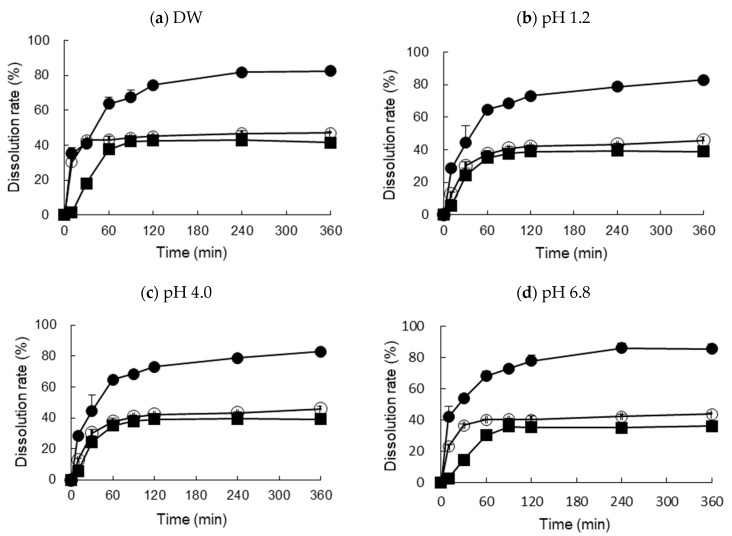
Dissolution profiles of optimized RXB-ASD, RXB powder, and commercial tablet (Xarelto 20 mg) in four different media (*n* = 3, means ± SD). (Key: ●: Optimized RXB-ASD, ○: Xarelto^®^ 20 mg, ■: RXB powder).

The commercial Xarelto^®^ (20 mg RXB) and RXB powders showed a relatively low dissolution of about 35–45% of the dose that dissolved after 120 min in distilled water (DW), SGF (pH 1.2), acetate buffer (pH 4.0), and SGF (pH 6.8), while the dissolution of the optimized RXB-ASD formulation reached approximately 80% after 120 min, which is more than a 2-fold change compared to the commercial Xarelto^®^ (20 mg RXB) and RXB powder (Figure 8). The optimized RXB-ASD formulation successfully improved the solubility of RXB. In addition, the similarity factor f2 in dissolution curves of Xarelto^®^ (20 mg RXB), RXB powder, and the optimized RXB-ASD formulation between water and SGF (pH 1.2) was 52.75, 69.15, and 73.74, respectively. The f2 values of those three between water and acetic acid buffer (pH 4.0) were 33.31, 57.15, and 60.92, respectively. The f2 values of those three between water and the SIF (pH 6.8) were 27.64, 60.37, and 59.45, respectively. Therefore, in terms of the capacity for overall dissolution, it is considered that the dissolution of the optimized RXB-ASD formulation would not be affected by the pH variation in vivo.

**Figure 9:**
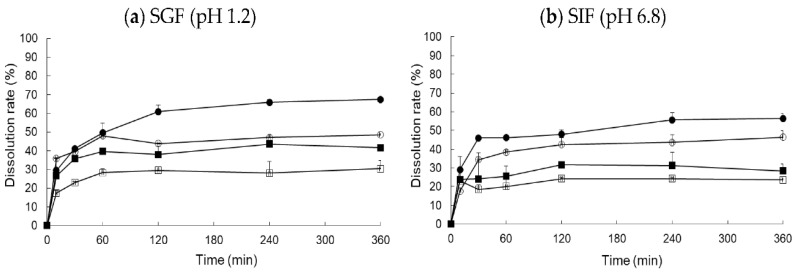
Food-effect dissolution rate of optimized RXB-ASD and a commercial tablet (Xarelto^®^, 20 mg) in simulated gastric fluid (SGF, pH 1.2) and simulated intestinal fluid (SGF, pH 6.8). (Key: ○: Optimized RXB-ASD in FaSSGF (or FaSSIF), □: Xarelto in FASSGF (or FaSSIF), ●: Optimized RXB-ASD in FeSSGF (or FeSSIF), ■: Xarelto in FeSSGF (or FeSSIF).

To monitor the food effect, the change in the dissolution of the formulations was monitored in in vitro dissolution tests in FaSSGF, FeSSGF, FaSSIF, and FeSSIF (Figure 9). As in the literature, we saw a 10–20% higher dissolution rate after ingestion, but high bioavailability can be expected because the dissolution rate was approximately 20% higher in the optimized formulation.

### 3.5. Stability

**Figure 10:**
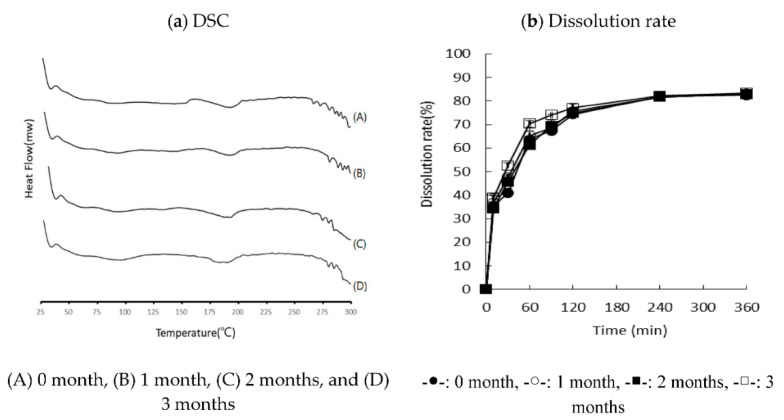
DSC and dissolution profile of optimized RXB-ASD for three months.

The optimized RXB-ASD was stored for three months under accelerated conditions to monitor the stability, which was assessed in terms of the crystallinity, physicochemical change, and dissolution rate. It showed the same endothermic behavior for three months without recrystallizing and exhibited excellent stability without a significant change in the dissolution rate (Figure 10).

### 3.6. Application to Pharmacokinetic Studies

The established analytical method was applied to a pharmacokinetic study following oral administration of 10 mg/kg RXB and RXB-ASD and intravenous administration of 2 mg/kg RXB to four male rats per group. The calibration curve exhibited linearity from 0.5 to 3000 ng/mL with a correlation of 0.0995 (Y = 0.014 x + 0.0002).

**Figure 11:**
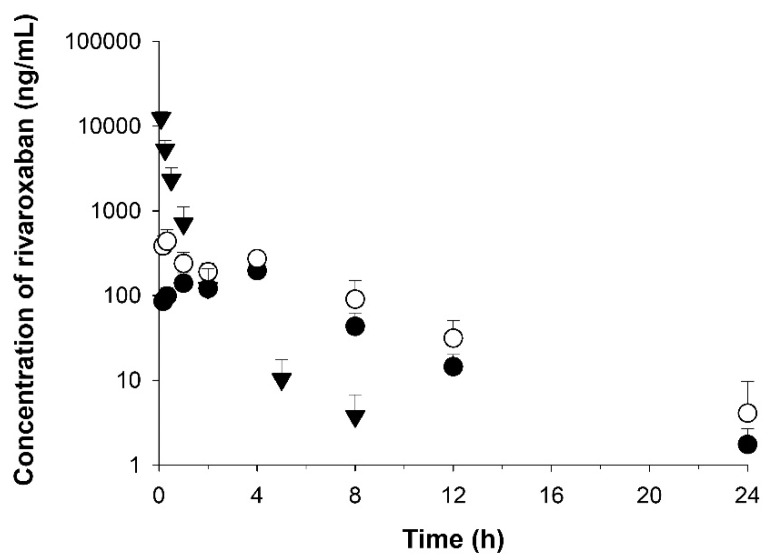
Temporal profiles of RXB concentrations in rat plasma after oral administration of optimized RXB-ASD (○) and RXB powder (●) at a dose of 10 mg/kg and after intravenous administration of RXB powder (▼). Data are shown as the mean ± SD (*n* = 4).

The mean plasma concentration vs. time profiles for RXB are shown in Figure 11. Following an oral gavage dose, in the group treated with the optimized RXB-ASD, the concentration rapidly increased and reached the peak concentration (C_max_) at 0.33 h (T_max_), with a mean value of 206.26 ng/mL (Table 6). Compared to after approximately 4 h in the group treated with the powder, RXB was very rapidly absorbed in the optimized RXB-ASD, indicating that it is possible to increase the systemic exposure of drugs with poor bioavailability. In addition, C_max_ and AUC_0–24 h_ in the group treated with the optimized RXB-ASD were 2.1-fold and 1.8-fold higher, respectively, than in the group treated with the powder. Around 4 h after oral administration, a double-peak phenomenon was observed, caused by entero–hepatic circulation, delayed gastric emptying, or absorption at various sites in the gastrointestinal tract [31]. In addition, the absorption process comprehensively describes the double-peaks of RXB plasma concentrations and the corresponding changes in the pharmacodynamic effect on prothrombin time in rats [32].

Comparing partial AUC_0–4 h_, the value of the optimized RXB-ASD was 1.8-fold higher than that of the powder formulation (HME, 1000 ng*h/kg vs. powder formulation, 548 ng*h/mL); comparing the partial AUC _4–24 h_, the value of the optimized RXB-ASD was 1.7-fold higher than that of the powder formulation (the optimized RXB-ASD, 1190 ng*h/kg, vs, powder formulation 696 ng*h/mL). At the 4-h time point, the partial AUC was equally separated with half of the total AUC_last_. This indicated that the absorption within 4 h (a short period) contributed to increasing the amount of the total AUC by 45% for the optimized RXB-ASD (AUC_0–4 h_, 1000 ng*h/mL; AUC_last_, 2180 ng*h) formulation, although the remaining 55% of the total AUC increased for 20 h, from 4 to 24 h (AUC_0–4 h_, 1190 ng*h/mL; AUC_last_, 2180 ng*h). The half-life (T_1/2_) and elimination rate constant (K_el_) were similar in both groups, at 3.1–3.2 h and 0.223–0.237 h, respectively. Although the mean residence time (MRT) was approximately 4.7–4.8 h, and a similar value was observed for both groups, the clearance (CL) value in the HME formulation was 0.58-fold that of the powder, and the V_d_ in the optimized RXB-ASD was 0.62-fold that of the powder. Considering CL and V_d_ values, the RXB of the HME formulation was not eliminated more rapidly than the powder. The bioavailability for RXB-ASD increased 1.8-fold to 8.6% compared with 4.9% for the RXB-powder.

Based on a pharmacokinetic study in rats, the results show that hot-melt extruded ASD containing RXB with combinations of polymers increased the absorption and bioavailability by improving the solubility in an in vivo animal study.

## 4. Conclusions

In this study, RXB-ASD was successfully prepared by HME technology using optimized compositions of PVA VA 64 and Cremophor^®^ RH 40. The in vitro drug dissolution study exhibited relatively high dissolution in four media and achieved about an 80% cumulative drug release in 120 min. The pharmacokinetic study of RXB-ASD in rats showed that the absorption was markedly increased in terms of the rate and amount, i.e., AUC and C_max_, compared to the raw RXB powder. This indicated that enhanced oral bioavailability can be obtained by our optimized RXB-ASD formulation.

## Figures and Tables

**Table 1:** Formulation of hot-melt extruded RXB-ASDs using various polymer mixtures.

Formulation	*w*/*w* Ratio						Content (%)	Dissolution Rate (%)
	RXB	Soluplus^®^	PVP VA 64	PVA	Cremophor^®^ RH 40	Gelucire^®^ 50/13		
F1	1	2	-	-	-	-	89.63 ± 3.31	24.52 ± 4.13
F2	1	4	-	-	-	-	90.56 ± 4.36	24.66 ±12.94
F3	1	-	2	-	-	-	94.05 ± 3.33	46.57 ± 0.57
F4	1	-	4	-	-	-	97.01 ± 1.24	51.89 ± 0.46
F5	1	2	2	-	-	-	91.26 ± 6.77	18.83 ± 4.32
F6	1	4	-	1	-	-	76.05 ± 2.23	52.72 ± 2.19
F7	1	4	-	-	1	-	92.48 ± 1.33	35.82 ± 6.61
F8	1	4	-	-	-	1	93.70 ± 1.48	33.44 ± 0.48
F9	1	-	4	1	-	-	73.78 ± 1.77	80.80 ± 1.16
F10	1	-	4	-	1	-	93.58 ± 1.26	83.29 ± 0.33
F11	1	-	4	-	-	1	95.38 ± 1.26	73.80 ± 3.76

**Table 2:** Preformulation experimental design of hot-melt extruded RXB-ASDs.

Independent Variables			Dependent Variables	
X1	X2	X3	Y1	Y2
1	0	180	92.32	48.55
1	0	200	101.52	41.17
1	0	220	94.75	33.90
1	10	180	97.01	44.46
1	10	200	99.49	43.00
1	10	220	96.82	42.30
1	20	180	107.36	40.88
1	20	200	97.98	42.71
1	20	220	88.12	45.27
2	0	180	88.09	48.96
2	0	200	86.67	51.30
2	0	220	91.29	75.32
2	10	180	97.71	44.82
2	10	200	101.77	43.91
2	10	220	95.68	77.17
2	20	180	94.13	44.41
2	20	200	90.45	49.01
2	20	220	101.68	80.51
4	0	180	95.40	60.62
4	0	200	97.76	64.29
4	0	220	105.12	73.36
4	10	180	94.81	53.18
4	10	200	105.81	68.05
4	10	220	97.63	84.40
4	20	180	93.35	50.96
4	20	200	98.53	79.89
4	20	220	96.31	87.40

X1, the PVP VA64 ratio; X2, Cremophor^®^ RH40 *w*/*w*%; X3, barrel temperature (°C); Y1, the content (%); Y2, the dissolution rate (%) in SIF (6 h).

**Table 3:** Solubility of RXB in 1% aqueous polymer solution (*n* = 3, means ± SD).

Polymer	Solubility (μg/mL)	Polymer	Solubility (μg/mL)
Distilled water	0.09 ± 0.00	HPC-L	5.79 ± 0.02
PVP VA 64	8.82 ± 0.16	Poloxamer 188	9.67 ± 0.09
PVP K90	6.32 ± 0.02	Solutol^®^ HS 15	12.45 ± 0.12
PVA	29.30 ± 8.76	Soluplus^®^	9.25 ± 0.04
PEG 200	12.38 ± 1.71	Gelucire^®^ 44/14	11.00 ± 0.02
PEG 4000	10.76 ± 0.21	Gelucire^®^ 50/13	14.23 ± 0.06
PEG 10000	8.97 ± 0.14	Labrasol^®^	6.02 ± 0.60
PEG 20000	9.02 ± 0.09	Cremophor^®^ RH 40	30.33 ± 5.24
HPMC 4M	6.47 ± 0.26	Cremophor^®^ EL	10.44 ± 1.51
HPMC 15M	5.12 ± 0.07	Tween 80	12.59 ± 1.07

**Table 4:** Experimental design of hot-melt extruded RXB-ASDs by CCD.

Independent Variable		Dependent Variable				
X1	X2	Y1	Y2	Y3	Y4	Y5
1.59	210	91.60	44.25	49.46	49.85	54.42
2	220	98.44	44.16	55.30	54.66	62.36
2	200	97.87	42.01	43.33	46.38	49.11
3	224.14	99.99	57.60	74.58	65.00	78.32
3	210	101.69	67.85	71.93	71.49	75.48
3	210	100.15	64.45	71.70	67.83	75.39
3	195.86	94.80	53.94	54.37	63.62	72.17
3	210	98.78	69.54	74.86	73.59	78.83
3	210	99.14	63.98	73.55	67.67	78.05
3	210	102.54	63.29	73.33	66.77	77.37
4	200	97.84	63.79	77.08	69.79	85.27
4	220	101.22	70.06	80.23	76.71	85.24
4.41	210	96.65	67.29	78.58	75.68	84.62

X1, the PVP VA64 ratio; X2, the barrel temperature (°C); Y1, the content (%); Y2, the dissolution rate (%) in SGF (2 h); Y3, the dissolution rate (%) in SGF (6 h); Y4, the dissolution rate (%) in SIF (2 h); Y5, the dissolution rate (%) in SIF (6 h).

**Table 5:** ANOVA results for all responses.

Variable	Parameter		
	*p*-Value	*R* ^2^	Lack of Fit
Y1: Content (%)	0.092	0.6820	0.165
X1: Polymer ratio	0.153		
X2: Barrel Temp.	0.110		
X1·X1	0.023		
X2·X2	0.388		
X1·X2	0.540		
Regression equation			
Y1 = −246 + 0.9X1 + 3.13X2 − 2.397X1·X1 − 0.000762X2·X2 + 0.070X1·X2			
Y2: Dissolution rate (%)—SGF, 2 h	0.000	0.9507	0.367
X1: Polymer ratio	0.000		
X2: Barrel Temp.	0.146		
X1·X1	0.002		
X2·X2	0.002		
X1·X2	0.506		
Regression equation			
Y2 = −2283 + 19.7X1 + 2177X2 − 5.22X1·X1 − 0.0522X2·X2 + 0.103X1·X2			
Y3: Dissolution rate (%)—SGF, 6 h	0.000	0.9628	0.018
X1: Polymer ratio	0.000		
X2: Barrel Temp.	0.002		
X1·X1	0.006		
X2·X2	0.008		
X1·X2	0.199		
Regression equation			
Y3 = −2184 + 86.3X1 + 19.54X2 − 4.59X1·1 − 0.0437X2·X2 − 0.221X1·X2			
Y4: Dissolution rate (%)—SIF, 2 h	0.001	0.9344	0.353
X1: Polymer ratio	0.000		
X2: Barrel Temp.	0.100		
X1·X1	0.017		
X2·X2	0.043		
X1·X2	0.353		
Regression equation			
Y4 = −1382 + 40.0X1 + 12.89X2 − 3.77X1·X1 − 0.0299X2·X2 − 0.034X1·X2			
Y5: Dissolution rate (%)—SIF, 6 h	0.000	0.9668	0.057
X1: Polymer ratio	0.000		
X2: Barrel Temp.	0.025		
X1·X1	0.005		
X2·X2	0.231		
X1·X2	0.046		
Regression equation			
Y5 = −866 + 107.8X1 + 6.99X2 − 4.22X1·X1 − 0.0136X2·X2 − 0.332X1·X2			

**Table 6:** Pharmacokinetic parameters of optimized RXB-ASD and raw RXB powder in rats after intravenous administration of 2 mg/kg and oral administration of 10 mg/kg. Data are shown as the mean ± SD (*n* = 4).

	Formulation (RXB-ASD)	Reference (RXB Powder)	
	P.O.	P.O.	I.V.
T_1/2_ (h)	3.10 ± 0.98	3.18 ± 0.98	1.21 ± 0.19
AUC_last_ (h·ng/mL)	2180 ± 455	1240 ± 170	5130 ± 1530
AUC_0–4 h_ (h·ng/mL)	1000 ± 257	548 ± 104	-
AUC_4–24 h_ (h·ng/mL)	1190 ± 295	696 ± 101	-
AUC_inf_ (h·ng/mL)	2210 ± 459	1250 ± 172	5140 ± 1530
MRT (h)	4.78 ± 0.74	4.73 ± 0.32	0.381 ± 0.093
T_max_ (h)	3.25 ± 1.50	0.33 ± 0.00	-
C_max_ (ng/mL)	436 ± 168	206 ± 26	-
V_d_ (mL/kg)	22,700 ± 6300	36,700 ± 2400	740 ± 267
CL (mL/h/kg)	4690 ± 1030	8130 ± 1100	420 ± 140
Kel (1/h)	0.237 ± 0.059	0.223 ± 0.039	0.583 ± 0.100
Bioavailability (%)	8.6	4.9	-

## Data Availability

Not applicable.
